# Complete Genome Sequences of vB_LmoS_188 and vB_LmoS_293, Two Bacteriophages with Specificity for *Listeria monocytogenes* Strains of Serotypes 4b and 4e

**DOI:** 10.1128/genomeA.00040-15

**Published:** 2015-04-09

**Authors:** Aidan Casey, Kieran Jordan, Aidan Coffey, Olivia McAuliffe

**Affiliations:** a Teagasc Food Research Centre, Moorepark, Fermoy, Co. Cork, Ireland; b Department of Biological Sciences, Cork Institute of Technology, Bishopstown, Co. Cork, Ireland

## Abstract

*Listeria monocytogenes* is responsible for the rare disease listeriosis, which is associated with the consumption of contaminated food products. We report here the complete genome sequences of vB_LmoS_188 and vB_LmoS_293, phages isolated from environmental sources and that have host specificity for *L. monocytogenes* strains of the 4b and 4e serotypes.

## GENOME ANNOUNCEMENT

*Listeria monocytogenes* is a Gram-positive facultative anaerobe and the causative agent of listeriosis, a disease associated with the consumption of contaminated food products. Its psychotropic nature, coupled with its ability to persist in the environment (1, 2), make it a serious food safety threat, manifested by the high mortality rates (20 to 30%) associated with listeriosis (3). Three serotypes of the species (1/2a, 1/2b, and 4b) are responsible for >90% of listeriosis cases, with serotype 4b strains associated with the greatest number of outbreaks (4). While the genomic diversity of *L. monocytogenes* has been well studied (5, 6), less is known about the genomic diversity of *L. monocytogenes* phages and, in particular, the genetic determinants responsible for the specific interactions between *Listeria* phages and their hosts.

Two *L. monocytogenes* bacteriophages were isolated from mushroom compost (vB_LmoS_293) and wild mushroom (vB_LmoS_188) samples. The genomes were sequenced by MWG Eurofins (Eurofins MWG Operon, Germany) on an Illumina MiSeq next-generation sequencing (NGS) system to >100× coverage. For each, sequencing yielded approximately 3 million reads, with an average length of 148 bp and an average quality score of 37. The removal of low-quality reads was undertaken using Trimmomatic (7), and overlapping paired-end reads were segregated using FLASH (8). The reads were assembled using the DNAStar Lasergene SeqMan NGen software (DNAStar, Inc., USA). Open reading frames (ORFs) were predicted using Glimmer version 3.02 (9) and RAST (10), and RAST was utilized in subsequent genome annotations. The annotations were verified and curated using BLASTp (11) and Artemis (12), while functional domains were predicted using InterPro (http://www.ebi.ac.uk/interpro).

The vB_LmoS_188 genome is 38,392 bp in length (G+C content, 35.9%), while bacteriophage vB_LmoS_293 is 40,759 bp in length (G+C content, 36.9%). PCR analyses confirmed that both genomes contain linear circularly permuted double-stranded DNA (dsDNA) with terminal redundancy. Sixty ORFs were detected in vB_LmoS_188, while 72 ORFs were detected in vB_LmoS_293. The ORFs predominantly begin with the ATG start codon (91.6% in vB_LmoS_188 and 87.5% in vB_LmoS_293). No tRNAs were detected. No function was assigned to 34/60 ORFs detected in vB_LmoS_188 or for 41/72 ORFs in vB_LmoS_293. The genomes are ordered in a modular fashion, consistent with previous observations for *Listeria* bacteriophages (13). Despite their similar host ranges and modular arrangements, the genomes of these two bacteriophages share only 37% nucleotide sequence identity and a maximum of 65% nucleotide identity with any other published *Listeria* phage genome in the NCBI database. These phages belong to a recently defined group of *Listeria* bacteriophages denoted orthocluster IV, along with phages A500, A118, A006, and LP-030-3 (14). Their specificity for *L. monocytogenes* strains of serotypes 4b and 4e is likely attributed to a small cluster of putative tail fiber genes, namely, ORFs 18 to 22 in vB_LmoS_188 and ORFs 19 to 23 in vB_LmoS_293, which are thought to function in bacterial host recognition.

### Nucleotide sequence accession numbers.

The genome sequences of these two phages have been deposited in GenBank under the accession numbers KP399677 (vB_LmoS_188) and KP399678 (vB_LmoS_293).
